# Investigating the effectiveness of mobilisation alarms to prevent hospital falls using disinvestment: A randomised clinical trial

**DOI:** 10.1016/j.ijnurstu.2025.105320

**Published:** 2025-12-15

**Authors:** Dai Pu, Kelly Stephen, Cassie McDonald, Jessica Baker, Georgina Sinforosa, Anastasia Hutchinson, Kerry Bradley, Kirsten Woods-Lyon, Michelle Tuck, Natasha Brusco, Lisa O’Brien, Debra Mitchell, Kate Steen, Melinda Webb-St. Mart, Peter Hunter, Philip L. Russo, Bernice Redley, Kelly-Ann Bowles, Mari Botti, Alison M. Hutchinson, Ronald Shorr, Terry Haines

**Affiliations:** a School of Primary and Allied Health Care, Faculty of Medicine, Nursing and Health Sciences, Monash University, Frankston, Victoria, Australia; b Eastern Health, Richmond, Victoria, Australia; c Alfred Health, Melbourne, Victoria, Australia; d Department of Physiotherapy, Faculty of Medicine, Dentistry and Health Sciences, The University of Melbourne, Carlton, Victoria, Australia; e Monash Health, Clayton, Victoria, Australia; f School of Nursing and Midwifery, Centre for Quality and Patient Safety Research, Institute for Health Transformation, Deakin University, Victoria, Australia; g Epworth HealthCare partnership, Richmond, Victoria, Australia; h Peninsula Health, Frankston, Victoria, Australia; i Rehabilitation, Ageing and Independent Living (RAIL) Research Centre, School of Primary and Allied Health Care, Monash University, Frankston, Australia; j School of Health Sciences, Department of Allied Health, Swinburne University of Technology, Melbourne, Victoria, Australia; k School of Translational Medicine, Monash University, Melbourne, Victoria, Australia; l School of Nursing & Midwifery, Monash University, Melbourne, Victoria, Australia; m Department of Nursing Research, Cabrini Institute, Malvern, Victoria, Australia; n Avondale University, Wahroonga, New South Wales, Australia; o School of Nursing and Midwifery, Deakin University, Geelong, Australia; p Health Complaint Commissioner, Melbourne, Victoria, Australia; q Barwon Health, Victoria, Australia; r College of Public Health and Health Professions, University of Florida, Gainesville, FL, USA; s College of Medicine, University of Florida, Gainesville, FL, USA; t North Florida/South Georgia Veterans Health System Geriatric Research Education and Clinical Center, Gainesville, FL, USA

**Keywords:** Falls, alarms, disinvestment, de-implementation, hospital

## Abstract

**Background:**

Mobilisation alarms are commonly used in hospitals to prevent falls in patients who are at high risk for falls, yet the evidence for their effectiveness is uncertain.

**Objective:**

To investigate the effectiveness of mobilisation alarms to prevent falls in hospitals.

**Design:**

This was a 3-group, concurrent, non-inferiority, stepped wedge, clinical trial with an embedded parallel, cluster randomised design that adopted a disinvestment approach. Disinvestment from the intervention was carried out from 1st April 2023 to 31st January 2024.

**Setting(s):**

This study was conducted in one private health service and four public health services in Metropolitan Melbourne, Australia.

**Participants:**

Acute and sub-acute hospital wards with at least a 3 % rate of use of mobilisation alarms. Twenty-two wards were screened and found eligible for the trial, 18 wards were recruited and randomised. A random subsample of patients in the recruited wards completed questionnaires.

**Methods:**

Two conditions were evaluated against the “current” condition of high alarm use (>3 %), a “reduced” rate of use of mobilisation alarms (<3 % but >0 %) and “eliminated” use of alarms (0 %). Rate of falls was the primary clinical outcome; data for a range of other measures were collected for secondary clinical and intervention outcomes.

**Results:**

There were 11 acute wards and 7 sub-acute wards with 157,037 occupied bed days observed; 1319 individual patients completed questionnaires. Non-inferiority of the “reduced” condition compared to the “current” condition was demonstrated [95 %, one-tailed, non-inferiority confidence limit of 2.00 falls/1000 occupied bed days (OBDs) increase] but not for the “eliminated” condition (3.68 falls/1000 OBDs increase). Superiority of any intervention condition was not demonstrated over another [“current” vs “reduced”: 0.22 falls/1000 OBDs (two-tailed, 95 % CI: −1.89 to 2.34), “current” vs “eliminated”: 0.90 (−2.41 to 4.21), “reduced” vs “eliminated”: 0.67 (−2.12 to 3.47)]. Patients’ frequency of sleep disturbance due to alarms was reduced in the “eliminated” condition [ordered logistic coefficient 0.47 (0.08 to 0.87)].

**Conclusions:**

Reduction in use of mobilisation alarms was not inferior to usual care, but complete elimination was uncertain.

**Registration:**

The trial was registered with the Australian New Zealand Clinical Trials Registry, trial ID: ACTRN12621000823875p (https://www.anzctr.org.au/Trial/Registration/TrialReview.aspx?ACTRN=12621000823875p). Registered on 28/06/2021, first enrolment on 26/10/2022.

## 1. Background

Mobilisation alarms are commonly used as a fall prevention strategy in hospitals (Brusco et al., 2021). They are intended to alert ward staff when a patient mobilises and may be at risk of falling. There are many products used, including pressure sensitive pads that can be placed on the bed, chair or on the floor next to the hospital bed, alarms attached to the patient that are triggered with movement, or infra-red and video monitors in the hospital room. Approximately 11 % of resources utilised in attempting to prevent falls in Australian hospitals have been estimated to be accounted for in the purchase, application and staff time responding to alarms (Mitchell et al., 2018). There is currently uncertainty as to whether mobilisation alarms are effective in reducing falls in hospitals.

Authors of a 2018 Cochrane review (Cameron et al., 2018) concluded there was uncertain evidence for alarm effectiveness in preventing falls based on three clinical trials that examined the use of mobilisation alarms in isolation (Shorr et al., 2012; Tideiksaar et al., 1993; Wolf et al., 2013). Randomised trials where mobilisation alarms have been used as a part of multifactorial intervention programs,(Barker et al., 2016; Cumming et al., 2008) a randomised trial excluded from the Cochrane review due to the primary outcome being bedside falls rather than total falls (Sahota et al., 2014), and a quasi-experimental study published since the Cochrane review (Visvanathan et al., 2022) have all shown no difference in falls outcomes. Further, observational research has identified that 52 % of alarm activations are false alarms, and there is a response time mean of 65 s if a nurse is not already in the room when the alarm is triggered (Brusco et al., 2021).

This evidence indicates that use of mobilisation alarms may be a form of low-value care. However, health services are likely to be reluctant to disinvest from their use without evidence that doing so will not increase the rate of falls in their health services. It is also important to understand whether completely eliminating the use of mobilisation alarms has a different effect to reducing their use. This clinical context lends itself to a non-inferiority research paradigm, where clinicians and managers may be satisfied to change use of an intervention as long as we have sufficient confidence that any negative consequences do not exceed a pre-specified threshold (the non-inferiority margin). This non-inferiority margin represents the costs and negative aspects of using that intervention expressed in terms of a clinical outcome (D’Agostino Sr. et al., 2003). The context of mobilisation alarm use in hospitals fits this paradigm well because there is evidence of costs and negative impacts from use of alarms which means that even if they are useful for preventing falls, that this level of impact would need to be beyond a certain threshold in order to justify their use. This study aimed to investigate whether “reduced” or “eliminated” mobilisation alarm conditions are non-inferior to “current” practice on wards with high levels of mobilisation alarm use for the prevention of falls; and whether there is superiority between these conditions for the prevention of falls.

## 2. Methods

### 2.1. Design

This was a 3-group, concurrent, non-inferiority, stepped-wedge trial with cluster randomisation. A detailed protocol has been published (Haines et al., 2021) which described our evaluation approach with both quantitative and qualitative elements. This paper reports on the quantitative components of the trial. The trial was registered with the Australian New Zealand Clinical Trials Registry. Trial ID: ACTRN12621000823875p; trial registration URL: https://www.anzctr.org.au/Trial/Registration/TrialReview.aspx?ACTRN=12621000823875p.

### 2.2. Participants and setting

This study was conducted in acute and sub-acute wards located across private and public health services in the State of Victoria, Australia. Disinvestment from the intervention was carried out from 1st April 2023 to 31st January 2024.

Inclusion criteria for wards were having a minimum of 20 beds, and >3 % usage of mobilisation alarm identified through a daily cross-sectional audit over a two-week period. Emergency, paediatric, mental health, and palliative care wards were excluded.

Ward sample size calculations were detailed in the published protocol (Haines et al., 2021). A subsample of patients on participating wards was approached to complete a questionnaire if they were planned for discharge within the next 36 h on the day of the weekly audit.

### 2.3. Intervention and control

There were three conditions in this trial:

- “Current”: Use of alarms remained unchanged (target: ≥3 % of occupied bed days [OBD] audited involved use of mobilisation alarms)
- “Reduced”: Use of alarms reduced (target: <3 % but >0 % of OBD audited involved use of mobilisation alarms)
- “Eliminated”: Use of alarms eliminated (target: 0 % of OBD audited involved use of mobilisation alarms).

There was no restriction on the types of mobilisation alarms that could be used.

### 2.4. Outcome measures

The primary outcome was rate of falls. Secondary outcomes were falls related injury, patient satisfaction with care (Draper et al., 2001), subjective sleep quality (Carpenter and Andrykowski, 1998), rate of newly developed pressure injuries, reported medication errors, and 30-day readmissions (Table 1). The frequency and use of other falls prevention strategies were also captured as potential confounding variables.

### 2.5. Procedure

Organisational leaders of health services who had an interest in the trial were asked to nominate potentially eligible wards and audit their use of mobilisation alarms for two weeks. Nurse Unit Managers of wards that met the inclusion criteria were invited to participate.

The 18 recruited wards were paired into 9 clusters and underwent a 2-step randomisation procedure to determine when they would transition from the current condition (step 1) and whether they would transition to a “reduced” or “eliminated” condition (step 2). The investigator conducting the randomisation (final author) used a computer-generated allocation (Microsoft Excel) and was blinded to ward/hospital identify through the use of pseudonyms. The trial began with all wards under their usual alarm use condition. Pairs of wards transitioned into the alarm use condition at the start of the calendar month they were randomised to, such that by the end of the trial, all wards would be in either the “reduced” or “eliminated” condition, with each ward spending different lengths of time under that condition.

In the “reduced” and “eliminated” conditions, staff were informed that they should adhere to their assigned condition as much as possible unless clinical judgement dictated that a mobilisation alarm *must* be used to ensure patient safety, and the alarm should be removed as soon as it was deemed safe to do so. Ward staff were allowed to self-determine how this would happen.

A non-inferiority margin was set by the project governance committee. Committee members were management and clinical representatives of participating sites. They were asked to discuss and decide what the minimum reduction in falls would be to justify the costs (financial and staff time) and other potential impacts on patient care (e.g. sleep disturbance) of using mobilisation alarms. This threshold was set at 2 falls/1000 OBD based on majority vote. This threshold also served as the safety stopping rule. Safety-monitoring checks were conducted every month, where the “reduced” and “eliminated” conditions’ fall rates were checked to determine if 95 % CIs were in excess of this margin compared to the “current” condition.

Prior to the start of the trial, two members of the research team visited each ward and gave a presentation on the evidence and motivation behind the research. The two researchers visited again in the month before each ward was due to begin its transition to inform the staff of the intervention condition they were randomised to, and for all staff to ask questions and express concerns. All ward staff were invited to attend these information sessions to promote acceptance and compliance with the trial conditions (Haines et al., 2017).

Weekly audits were conducted across the trial period at each site. Researchers visually inspected patient rooms weekly to gather data on fall prevention strategies including mobilisation alarm use. Additional twice weekly communications were held with nursing to confirm number of alarms in use and if any falls had occurred. These researchers also approached those patients planned for discharge within 36 h for consent to complete questionnaire-based outcomes.

### 2.6. Implementation of alarm use changes

Strategies aligned with the Expert Recommendations for Implementing Change (ERIC) taxonomies (Powell et al., 2015) were utilised to promote acceptance and adherence to the trial conditions. These were:

- Formation of a project governance committee with management and clinical representatives from participating sites.
- Involvement of executive team members through internal communications between committee representatives and executive members. Where requested, project lead researchers also met with members of health service executive.
- Provision of pre-workshops for front-line staff. These involved provision of information about the trial, information about the evidence regarding the effectiveness of bed alarms to date, information about the pilot work undertaken by the team, and opportunity for discussion of these topics. These meetings invited participation from nursing, medical and allied health staff on each participating ward. One or two workshops per ward were provided, with the additional workshop provided upon local request.
- Written communications were provided to nurse unit managers following the workshop for local distribution. A local champion to implement the change was identified, and began developing local strategies for how the change in practice would be implemented. Health service nurse educators were identified to support the local unit managers to develop strategies.
- Pre-implementation (change in practice) workshops were provided in the month before the scheduled transition in practice. Changes that were about to occur during the following period were discussed. Staff confidence and potential barriers to implement these were discussed, along with locally developed strategies for implementation. Local strategies included introduction of gatekeeper strategies (permissions were required from Nurse Unit Manager before an alarm could be used), physical removal of alarms from storage areas, and promotion of alternate falls prevention strategies.
- During the intervention period, the study auditor would notify the local site champion if a greater number of alarms was identified on the ward and remind them to reduce the number of alarms when they considered this was possible.

### 2.7. Analysis

The difference in mean rate of falls was compared between conditions (“current” vs “reduced” vs “eliminated”) based on data from the entire trial at the summative ward-month level (i.e. one unit of measure per ward per month) using a mixed-effects, general linear model. Ward and cluster (pair) were entered as random effects, with calendar month entered as a categorical fixed effect consistent with recommendations for analysis of stepped-wedge trials (Hemming et al., 2015). Classifications of inferiority/non-inferiority/uncertainty of inferiority were made relative to our one-tailed, 95 % confidence, non-inferiority margin of 2 falls per 1000 OBD. All pair-wise between-condition contrasts were examined for superiority using two-tailed 95 % confidence intervals. Visual investigations of falls data were undertaken using “transition-relative” line graphs of both raw data (Supplementary Figs. 1a and 2a) and residuals post analysis of a mixed model including calendar month (fixed) and ward (random) (Supplementary Figs. 1b and 2b) (Haines and Hemming, 2018). These analyses indicated that potential analyses specified in the protocol investigating growth or decline in intervention effect over time were not indicated. Secondary outcomes of patient satisfaction and sleep disturbance questions that were collected from a sub-sample of patients were analysed using a mixed-effects logistic model for ordered responses with data at the patient level rather than summative ward-month level. Secondary clinical outcomes and nonfalls related adverse events were compared as a change in rate of events, or difference in proportion of patients with the outcome when audited before and after the transition of the wards from “current” to the “reduced” or “eliminated” condition (Table 3).

### 2.8. Deviation from protocol

The secondary outcome of “Rate of hospital readmission within 30 days” could not be examined as we were unable to disaggregate readmissions data from individuals who may have been exposed to multiple different wards participating in this study within the same admission. Analyses comparing fall rates between conditions using incident rate ratios were added to enable future meta-analyses with similar trials that have analysed data in this way.

Difficulty satisfying assumptions of comparing primary and some secondary outcomes between groups using the pre-planned Gaussian (normal) distributional family were encountered. A Poisson distributional family with log link function was employed for count/rate outcomes expressed as frequencies of events per month adding an exposure variable for the number of OBDs for that ward in that month. These analyses employed robust standard errors and the Delta method for calculating standard errors when contrasts were expressed as differences in rates rather than incidence rate ratios. Non-parametric bootstrap resampling was used to generate confidence intervals for differences in outcomes expressed as proportions of patients audited (Barber and Thompson, 2000).

Pre-trial stakeholder feedback led to audit items i) family members being present, ii) crash mat on floor beside bed, iii) phone within reach, and iv) table within reach being added to the weekly audit before trial commencement, while v) locked bed, and vi) anti-slip floor mat were removed.

## 3. Results

**Fig. 1.**
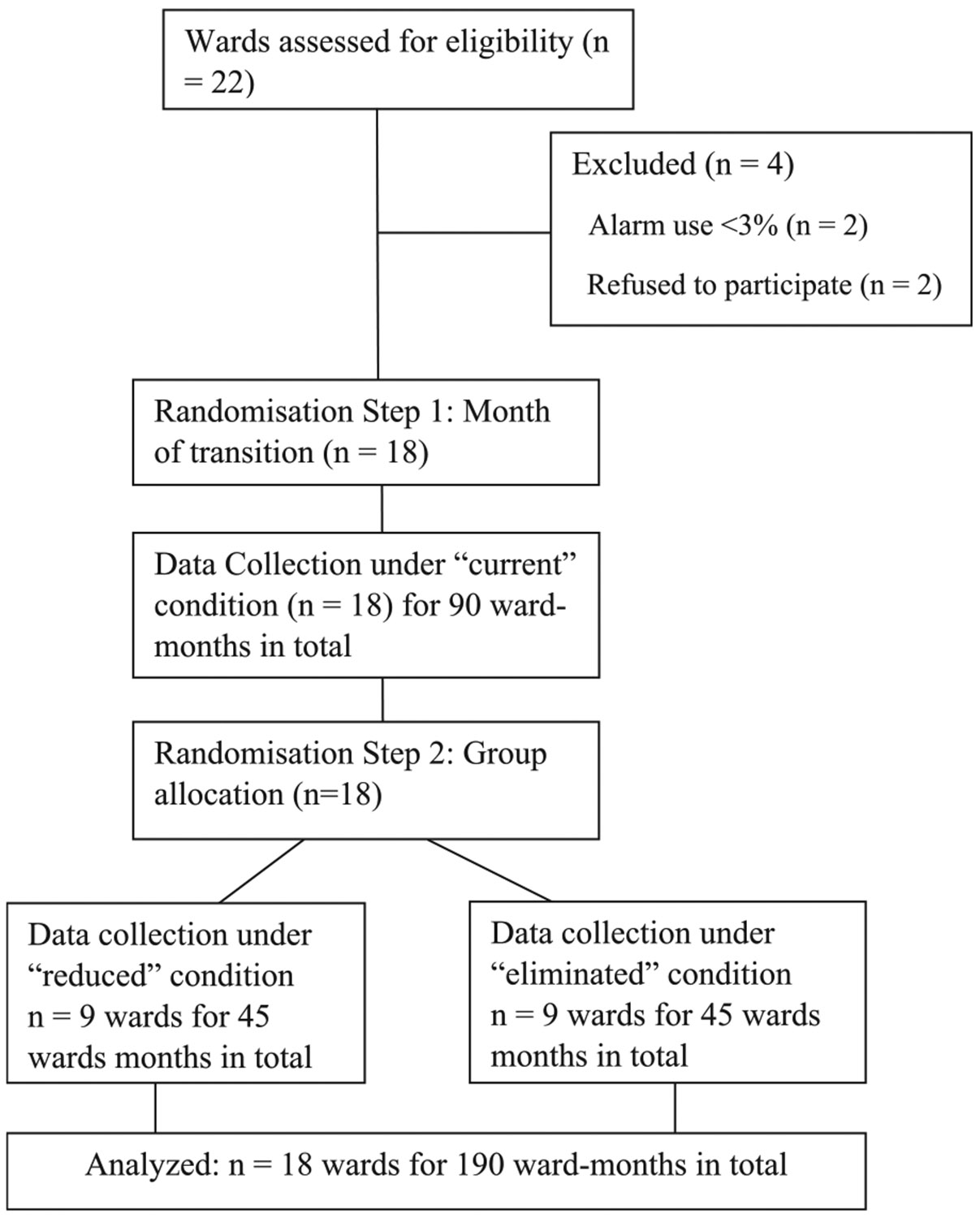
CONSORT flow chart of ward recruitment for this trial.

Twenty-two wards were audited for eligibility, and 18 were recruited (Fig. 1). There were 7 wards from one private health service and 11 wards from 4 public health services, all located in Melbourne, Victoria, Australia (Table 2). The trial commenced 1st April 2023 and concluded 31st January 2024 (10 calendar months). Pre-trial alarm use, ward type, and bed capacity/occupancy during the trial period are presented (Table 2). All wards used pressure-sensor triggered alarms placed on the bed or chair, which had the capacity to make an audible alarm and/or notify via pager carried by a nurse. Two wards (labelled 4 and 17 in Table 2) also had additional access to in-built alarms that were part of the hospital beds. Monthly safety monitoring did not find a breach of the safety stopping rule at any point during the trial (Supplementary eTable 1).

A total of 2522 individual patients were identified to complete a questionnaire about their stay; 625 (24.78 %) were discharged before they could be approached, 69 (2.74 %) declined to participate, and 509 (20.18 %) were unable to participate due to being away from their bedspace at the time of data collection or reduced cognitive capacity. A total of 1319 (52.30 %) individual patients completed questionnaires for the secondary outcomes of patient satisfaction with care and sleep quality. They had a mean age of 69.62 years (range = 19–101, standard deviation = 16.27), 59.21 % of whom were female (n = 781). The patients had a mean hospital length-of-stay of 8.52 days (range = 0–308, standard deviation = 15.88). There were 702 (53.22 %) patients who completed the questionnaires while their ward was under the “current” condition of alarm usage, 248 (18.80 %) patients completed the questionnaires under the “reduced” condition, and 369 (27.98 %) patients completed the questionnaires under the “eliminated” condition.

### 3.1. Primary clinical outcome

Non-inferiority of the “reduced” condition compared to the “current” condition was demonstrated as the 95 %, one-tailed, non-inferiority confidence limit of a 2.00 falls/1000 OBDs increase just fell below the 2.00 falls/1000 OBDs non-inferiority threshold set for this trial. Non-inferiority of the “eliminated” condition compared to the current condition was not demonstrated as the 95 %, one-tailed, non-inferiority confidence limit was an increase of 3.68 falls/1000 OBDs.

Pairwise comparisons of superiority (Table 3) examining the difference in mean rates of falls showed that the “current” condition was not superior to the “reduced” condition, or the “eliminated” condition. There was also no difference in superiority analyses between the “reduced” or “eliminated” conditions. Incident rate ratios of these comparisons are also presented (Supplementary Table 2).

### 3.2. Secondary outcomes

No differences were found before and after transition for most secondary outcomes with the exception of patient-reported sleep disturbance due to mobilisation alarms, with patients in wards that “eliminated” mobilisation alarms reporting less sleep disturbance (Table 3).

### 3.3. Intervention contamination

The rates of use of other falls prevention strategies that were not mobilisation alarms varied during the trial (Table 3). Patients were more frequently positioned to be visible from outside the room during the “reduced” condition compared to the “current” condition. Patients in the “eliminated” condition were less likely to be observed to have their call bell, table, and phone within reach. Those in the “reduced” condition were less likely to have their table within reach.

### 3.4. Intervention fidelity

Raw data for intervention fidelity are presented (Supplementary Table 3). Bootstrap analyses indicated there was a significant reduction in the proportion of patients with a mobilisation alarm on wards that transitioned to the “reduced” condition [absolute reduction 7.6 % (95 % CI: 4.9 % to 10.6 %)] and on wards that transitioned to the “eliminated” condition [absolute reduction 10.3 % (95 % CI: 6.5 % to 14.4 %)]. However, there were inconsistencies across wards. Despite all wards exceeding the 3 % alarm use threshold during the 2-week, pre-study eligibility audit, two wards did not exceed the 3 % alarm use threshold in any month of observation during their “current” condition period (one assigned to “reduced”, one assigned to “eliminated”). The “reduced” condition had 22 ward-months out of 45 where the alarm use rate was both <3 % but not 0 %. The “eliminated” condition had 29 ward-months out of 45 with 0 %.

## 4. Discussion

This trial identified that reducing use of mobilisation alarms was not inferior to usual care, but it remains uncertain as to whether complete elimination is non-inferior to usual care. This non-inferiority finding of reducing mobilisation alarm use was marginal (the confidence margin was only just below the threshold) and relative to the pre-specified, non-inferiority margin established by stakeholders from our participating sites. It is possible that stakeholders from other sites may set a lower non-inferiority margin which may impact the applicability of our result to their setting. This trial experienced larger than anticipated standard deviations in monthly fall rates. This study was undertaken during the tail-end of the COVID-19 pandemic. Ongoing outbreaks of COVID-19 and other infectious diseases on the wards may have contributed to this higher than expected variability as they have been found to be associated with higher rates of falls on hospital wards (Fernandez et al., 2025; Liang et al., 2021).

Our findings of uncertainty regarding the benefits of using mobilisation alarms for the prevention of falls are consistent with recommendations and findings from recent World Guidelines for Preventing Falls in older adults and systematic review meta-analyses (Montero-Odasso et al., 2022; Morris et al., 2022). Mobilisation alarms in their earliest form were first investigated in 1993 in a trial of 70 patients, which did not find any statistically significant effects for their use but were well accepted by nursing staff, patients and their families (Tideiksaar et al., 1993). Since then, trials have been conducted investigating mobilisation alarms in isolation (Shorr et al., 2012) and in combination with other interventions (Barker et al., 2016; Cumming et al., 2008). Not one of these trials has reported a reduction in falls as a result of their intervention. Part of the challenge may lie in limited nursing staff availability to respond to alarms (Marć et al., 2019). Observational research indicates it takes 65 s for an alarm to be responded to if a nurse is not already in the room when an alarm is triggered (Brusco et al., 2021). Considering our trial findings in the context of previous research further indicates that the staff time and financial resources required to use mobilisation alarms to prevent falls may not be justified, particularly given negative impacts on patient sleep, mobility, autonomy and patient preferences (Brusco et al., 2021; Growdon et al., 2017; Radecki et al., 2018).

Previous studies have identified alarms sounding at night may be sources of sleep disturbance for patients (Delaney et al., 2018; Schoen et al., 2016; Stephen and Campbell, 2024), nurse medication errors associated with distractions (Bucknall et al., 2019) and fatigue (Gündoğan and Erdağı Oral, 2023) from alarms. The impact of mobilisation alarms on patient sleep disturbance was demonstrated in the present study, the first time an experimental study has done so. This has implications for the recovery of hospitalised patients as sleep is integral to the healing process (Hillman, 2021), and disturbed sleep is associated with complications such as delirium (Miller, 2015; Sangari et al., 2021), which can increase the risk of falls (He et al., 2022; Lakatos et al., 2009; Sillner et al., 2019). However, this study did not find changes in reported medication errors in association with alarm reduction or elimination.

The use of falls prevention strategies other than mobilisation alarms was documented as possible contaminations in this research. Our data indicated only minor changes in the use of these falls prevention interventions. Strategies that did appear to change [the placement of frequently used items within reach of the patient (phone, table, call bell) being significantly reduced], would arguably indicate that a weaker falls prevention approach overall was being used during the “eliminated” condition. It should further be noted that these potentially contaminating interventions have very little evidence supporting their effectiveness (Morris et al., 2022).

## 5. Limitations

This trial was challenged by limitations with intervention fidelity with some wards having lower than anticipated rates of mobilisation alarm use during the “current” condition (planned to be >3 %), and also some wards having outside target range use of alarms in the intervention conditions. Despite this, there was a substantial, immediate, and sustained reduction in mobilisation alarm use in many wards. For other wards, it should be noted that the COVID-19 pandemic led to a delay between the pilot work conducted in 2019 (Brusco et al., 2021) and the commencement of the trial in 2023. There was turn-over in a number of the staff that we had worked with from the beginning of this process, as a result, momentum may have been lost for health service staff to adopt and adhere to the trial conditions on these wards. Hospital wards have complex environments in which leadership, culture and practice can be dynamic and change across time. These factors can all influence the readiness of a ward to adopt (or not) practice changes in-line with the research conditions, and the variability in how wards reduced and/or eliminated mobilisation alarms may be reflective of the mixed impacts of these factors. Despite this, there was a substantial reduction in alarm use between the conditions as planned, providing a useful intervention signal to address our research questions.

The mobilisation alarms used in this trial varied to some extent between participating wards, but all involved a pressure sensitive detection system. It is possible that alarm systems that use other types of detection systems [e.g. Accelerometer (Visvanathan et al., 2022), infrared beam(Banerjee et al., 2003), or imaging-based detection systems (Cournan et al., 2018; Ergai et al., 2024)] may generate different results, particularly if they can shorten the amount of time in which a staff member can be present to assist the patient without increasing the rate of false-positive alarm activations (Brusco et al., 2021).

## 6. Conclusions

Reducing use of mobilisation alarms on hospital wards with high rates of use was non-inferior to usual care. It was uncertain as to whether complete elimination was non-inferior to usual care and whether use of mobilisation alarms reduces the rates of falls. There was less reported disturbance to patient sleep due to alarms when the alarm use was eliminated, but this was not reflected in improved perceived sleep quality overall. This research adds to the body of research indicating that routine use of mobilisation alarms may represent a form of low-value care.

## Supplementary Material

Supplementary Material

## Figures and Tables

**Table 1:** Outcome measures and corresponding data collection approaches.

Outcome	Outcome type	Data collection approach(es)
Rate of Falls	Primary clinical outcome	1. Extraction from hospital records 2. Interview with nurse unit managers (NUM)
Rate of falls-related injuries	Secondary clinical outcome	Extraction from hospital records
Patient satisfaction with care	Secondary clinical outcome	Victorian Patient Satisfaction Survey (Draper et al., 2001) with patient sub-sample
Patient sleep quality	Secondary clinical outcome	Questions from the Pittsburgh Sleep Quality Index (Carpenter and Andrykowski, 1998) with patient sub-sample
Rate of newly developed pressure injuries (since admission to ward)	Secondary outcome - Non-falls related adverse events	Extraction from hospital records
Rate of reported medication error	Secondary outcome - Non-falls related adverse events	Extraction from hospital records
Rate of hospital readmission within 30 days	Secondary outcome - Non-falls related adverse events	Extraction from hospital records
Proportion of patients with mobilisation alarms	Secondary outcome – Intervention fidelity	Weekly direct observations of ward beds
Rate of use of “other” confounding falls-prevention interventions	Secondary outcome – Intervention contamination	Weekly direct observations of ward beds

**Table 2:** Health services and wards recruited to this trial.

Health service	Hospital	Ward	Ward type	Bed capacity (mean occupancy)	Pre-trial alarm use rate (%)
A	i	1	Sub-acute	30 (29.3)	4.5
	ii	2	Acute	20 (19.6)	4.42
B	iii	3	Acute	27 (19.4)	12.27
	iv	4	Acute	24 (24)	9.58
C	v	5	Acute	32 (32)	11.34
	vi	6	Sub-acute	34 (34)	3.28
	vii	7	Acute	33 (33)	7.94
	vii	8	Acute	36 (36)	3.99
	viii	9	Acute	32 (32)	3.57
	viii	10	Acute	34 (34)	4.01
	viii	11	Acute	39 (39)	6.08
D	ix	12	Sub-acute	30 (29.9)	20.45
	x	13	Acute	36 (36)	4.44
	xi	14	Sub-acute	29 (27.75)	16.66
	xi	15	Sub-acute	32 (26)	24.35
E	xii	16	Acute	30 (29.5)	3.39
	xiii	17	Sub-acute	30 (29.5)	14.24
	xiv	18	Sub-acute	30 (29.5)	10.18

**Table 3:** Primary and secondary outcomes and comparisons between groups.

Outcomes	Current condition	Reduced condition	Eliminated condition	Difference between groups (superiority analysis, 2-tailed 95 % CI)			
			
	Mean (sd), median (IQR)			Current vs. reduced	Current vs. eliminated	Reduced vs. eliminated	
**Primary clinical outcome**							Differences in rates
Rate of falls/1000 OBD^a^	9.94 (7.15), 8.33 (5.13 to 13.10)	11.15 (6.97), 9.52 (5.38 to 15.17)	10.31 (6.79), 8.32 (6.12 to 12.32)	0.22 (−1.89 to 2.34), *p* = 0.84	0.90 (−2.41 to 4.21), *p* = 0.60	0.67 (−2.12 to 3.47), *p* = 0.64	
**Secondary clinical outcome**							
Rate of falls-related injuries/1000 OBD^a^	2.53 (4.60), 1.42 (0 to 3.34)	2.43 (2.93), 1.08 (0 to 4.06)	2.12 (2.39), 1.11 (0 to 3.40)	0.67 (−0.29 to 1.64), *p* = 0.17	0.75 (−0.19 to 1.68), *p* = 0.12	0.07 (−0.67 to 0.81), *p* = 0.84	
Patient satisfaction with care^c^	4.63 (0.73), 5 (4 to 5)	4.69 (0.59), 5 (4.25 to 5)	4.63 (0.73), 5 (4 to 5)	−0.34 (−0.81 to 0.14), *p* = 0.16	−0.32 (−0.76 to 0.12), *p* = 0.16	0.02 (−0.36 to 0.40), *p* = 0.91	Coefficients based on ordered logistic model
Patient sleep quality^d^	2.78 (0.87), 3 (2 to 3)	2.78 (0.88), 3 (2 to 3)	2.73 (0.86), 3 (2 to 3)	0.31 (−0.08 to 0.70), *p* = 0.12	0.17 (−0.19 to 0.52), *p* = 0.36	−0.14 (−0.49 to 0.21), *p* = 0.42	
Patient sleep disturbance from alarms^e^	3.03 (1.11), 3 (2 to 4)	3.00 (1.14), 3 (2 to 4)	3.12 (1.10), 4 (3 to 4)	0.40 (−0.01 to 0.82), *p* = 0.06	0.47 (0.08 to 0.87), *p* = 0.02	0.07 (−0.31 to 0.45), *p* = 0.71	
**Secondary outcome - Non-falls related adverse events**							Differences in rates
Rate of newly developed pressure injuries/1000 OBD^a^	1.74 (2.40), 1.12 (0 to 2.22)	1.32 (1.29), 1.08 (0 to 2.13)	1.21 (1.17), 1.08 (0 to 2.11)	−0.29 (−1.28 to 0.70), *p* = 0.56	−0.40 (−1.26 to 0.46), *p* = 0.36	−0.11 (−0.61 to 0.39), *p* = 0.67	
Rate of reported medication errors/1000 OBD^a^	4.98 (3.64), 3.86 (2.30 to 7.08)	3.78 (2.47), 3.07 (2.10 to 5.34)	3.30 (2.13), 2.95 (1.90 to 4.66)	−0.12 (−0.86 to 0.62), *p* = 0.76	−0.58 (−1.57 to 0.40), *p* = 0.25	−0.47 (−1.86 to 0.93), *p* = 0.51	
**Secondary outcome – Intervention contamination**							
Falls alert signs/ward^a^	22.86 (16.94), 24.13 (8.00 to 28.75)	24.47 (11.55), 24.85 (18.33 to 28.98)	24.49 (10.7), 24.75 (19 to 27.25)	1.02 (−4.12 to 6.15), *p* = 0.70	3.88 (−2.24 to 10.01), *p* = 0.21	2.87 (−4.84 to 10.57), *p* = 0.47	
Family member present^b^	0.11 (0.06), 0.11 (0.07 to 0.15)	0.13 (0.06), 0.13 (0.08 to 0.18)	0.13 (0.06), 0.13 (0.09 to 0.19)	−0.01 (−0.04 to 0.02)	−0.01 (−0.04 to 0.01)	−0.00 (−0.03 to 0.03)	Differences in proportions
Continuous patient observer^b^	0.02 (0.03), 0.01 (0 to 0.03)	0.01 (0.01), 0 (0 to 0.01)	0.01 (0.01), 0.01 (0.00 to 0.02)	−0.00 (−0.01 to 0.00)	0.00 (−0.01 to 0.01)	0.00 (−0.00 to 0.01)	
Regular bed in low position^b^	0.43 (0.32), 0.55 (0.01 to 0.72)	0.57 (0.21), 0.65 (0.48 to 0.70)	0.62 (0.24), 0.67 (0.58 to 0.75)	−0.02 (−0.09 to 0.05)	0.01 (−0.05 to 0.06)	0.03 (−0.03 to 0.08)	
Floor level bed^b^	0.04 (0.06), 0 (0 to 0.08)	0.06 (0.06), 0.06 (0 to 0.10)	0.04 (0.05), 0.01 (0 to 0.05)	0.01 (−0.01 to 0.03)	−0.01 (−0.03 to 0.01)	−0.02 (−0.04 to 0.01)	
Crash mat on floor beside bed^b^	0.03 (0.05), 0 (0 to 0.06)	0.05 (0.05), 0.04 (0 to 0.09)	0.03 (0.05), 0 (0 to 0.06)	0.01 (−0.00 to 0.03)	0.01 (−0.01 to 0.02)	−0.01 (−0.03 to 0.01)	
Patient visible from outside room^b^	0.42 (0.18), 0.44 (0.34 to 0.55)	0.44 (0.19), 0.44 (0.31 to 0.55)	0.45 (0.18), 0.47 (0.36 to 0.59)	0.08 (0.02 to 0.13)	0.05 (−0.01 to 0.10)	−0.03 (−0.08 to 0.01)	
Call bell within reach^b^	0.67 (0.12), 0.70 (0.62 to 0.74)	0.64 (0.11), 0.63 (0.57 to 0.70)	0.62 (0.07), 0.63 (0.59 to 0.66)	−0.03 (−0.08 to 0.02)	−0.06 (−0.11 to −0.01)	−0.03 (−0.08 to 0.02)	
Table within reach^b^	0.66 (0.13), 0.68 (0.60 to 0.75)	0.65 (0.11), 0.66 (0.57 to 0.71)	0.63 (0.08), 0.64 (0.57 to 0.68)	−0.01 (−0.06 to 0.03)	−0.07 (−0.13 to 0.02)	−0.06 (−0.11 to −0.01)	
Phone within reach^b^	0.50 (0.19), 0.53 (0.41 to 0.64)	0.48 (0.17), 0.44 (0.40 to 0.60)	0.50 (0.15), 0.53 (0.36 to 0.63)	−0.04 (−0.09 to 0.01)	−0.06 (−0.11 to −0.01)	−0.02 (−0.07 to 0.03)	
Bedside commode^b^	0.09 (0.07), 0.07 (0.04 to 0.13)	0.12 (0.05), 0.12 (0.08 to 0.15)	0.16 (0.13), 0.11 (0.06 to 0.22)	0.02 (−0.00 to 0.05)	−0.00 (−0.04 to 0.03)	−0.03 (−0.06 to 0.01)	
Ambulatory aid within reach^b^	0.19 (0.14), 0.16 (0.10 to 0.28)	0.24 (0.11), 0.23 (0.15 to 0.30)	0.23 (0.12), 0.20 (0.13 to 0.34)	0.02 (−0.03 to 0.07)	−0.00 (−0.05 to 0.04)	−0.02 (−0.08 to 0.04)	
Physical restraint^b^	0.01 (0.02), 0 (0 to 0.01)	0.01 (0.02), 0 (0 to 0.01)	0.01 (0.02), 0 (0 to 0.01)	−0.01 (−0.02 to 0.00)	−0.01 (−0.02 to 0.00)	0.00 (−0.01 to 0.01)	
Non-slip footwear available^b^	0.15 (0.11), 0.14 (0.05 to 0.22)	0.19 (0.16), 0.14 (0.04 to 0.33)	0.18 (0.15), 0.15 (0.04 to 0.29)	0.03 (−0.01 to 0.07)	0.01 (−0.03 to 0.05)	−0.02 (−0.06 to 0.02)	
Non-slip socks available^b^	0.08 (0.06), 0.08 (0.04 to 0.12)	0.04 (0.04), 0.04 (0.01 to 0.06)	0.04 (0.05), 0.02 (0.01 to 0.07)	0.01 (−0.02 to 0.03)	0.00 (−0.02 to 0.03)	−0.00 (−0.03 to 0.02)	

a Rate-based outcomes compared between conditions using mixed effect generalised linear model with Poisson distributional family and log link function.

b Proportions compared between conditions using the non-parametric bootstrap (2000 replications, percentile approach).

c Item wording: Overall, how would you rate the care you received while in hospital? Very good = 5, Good = 4, Average = 3, Poor = 2, Very poor = 1.

d Item wording: During your hospital stay, how would you rate your sleep quality overall? Very good = 4, Fairly good = 3, Fairly bad = 2, Very bad = 1.

e Item wording: How often was your sleep disturbed by alarms on the ward? Did not happen = 4, Some nights = 3, Most nights = 2, Every night = 1.

## Data Availability

Data are available from https://bridges.monash.edu/ndownloader/files/59307983.

## What is already known

- Mobilisation alarms are commonly used in hospitals to prevent falls in patients, yet the evidence for their effectiveness is uncertain, making alarms a potentially low-value care practice.
- Disinvestment from low-value care requires evidence to support their removal or reduction.

## What this paper adds

- Reduction in use of mobilisation alarms is not inferior to usual care, but uncertainty remains around complete elimination.
- High use of alarms did not lead to significantly lower fall rates compared to reduced or eliminated use.

## Appendix A. Supplementary data

Supplementary data to this article can be found online at https://doi.org/10.1016/j.ijnurstu.2025.105320.
