# Supplementary material for: Investigating the effectiveness of mobilisation alarms to prevent hospital falls using disinvestment: A randomised clinical trial
Source: Int J Nurs Stud. Author manuscript; Available in PMC 2026 Jan 23. (PMC12825039; doi:10.1016/j.ijnurstu.2025.105320)
Supplement: Supplementary Material [file NIHMS2133245-supplement-Supplementary_Material.docx]

**Supplementary Table 1. Monthly falls rates (falls/1000 OBD) at each ward across the period of the trial.**

**Supplementary Table 2. Incident rate ratio of outcome measures between groups.**

**Supplementary Table 3. Monthly mean rate of alarm use across the 18 wards during the 1-month trial period.**

**Supplementary Figure 1a: Transition-relative line graph of raw number of falls for wards in the “reduced” condition.**

**Supplementary Figure 1b: Transition-relative line graph of residuals for wards in the “reduced” condition.**

**Supplementary Figure 2a: Transition-relative line graph of raw number of falls for wards in the “eliminated” condition.**

**Supplementary Figure 2b: Transition-relative line graph of residuals for wards in the “eliminated” condition.**

| **eTable 1: Monthly falls rates (falls/1000 occupied bed days) at each ward across the period of the trial.** | | | | | | | | | | | | | | | | |
| --- | --- | --- | --- | --- | --- | --- | --- | --- | --- | --- | --- | --- | --- | --- | --- | --- |
|  |  | | 2023 | | | | | | | | | | | | 2024 | |
| Time | Ward | | April | | May | June | | July | | August | September | October | November | December | January | |
| Cluster 1 | 14 | | 9.22 | | 11.15 | 28.40 | | 12.29 | | 14.46 | 12.64 | 17.84 | 6.90 | 17.80 | 6.69 | |
|  | 15 | | 13.67 | | 18.42 | 25.32 | | 9.09 | | 21.19 | 12.64 | 7.11 | 14.77 | 14.57 | 25.51 | |
| Cluster 2 | 13 | | 2.89 | | 10.37 | 7.60 | | 6.49 | | 3.68 | 3.78 | 8.38 | 5.67 | 14.73 | 4.63 | |
|  | 12 | | 14.48 | | 9.68 | 12.26 | | 13.99 | | 11.83 | 20.09 | 15.09 | 15.70 | 24.73 | 14.02 | |
| Cluster 3 | 7 | | 10.80 | | 8.55 | 5.41 | | 12.35 | | 8.32 | 7.89 | 9.31 | 8.68 | 2.53 | 12.31 | |
|  | 5 | | 7.61 | | 9.25 | 11.53 | | 5.31 | | 6.33 | 4.35 | 6.32 | 12.92 | 9.52 | 5.41 | |
| Cluster 4 | 9 | | 5.39 | | 9.64 | 8.57 | | 3.09 | | 11.33 | 7.58 | 8.24 | 6.41 | 6.38 | 5.12 | |
|  | 10 | | 6.04 | | 4.23 | 3.28 | | 3.22 | | 4.12 | 13.97 | 7.50 | 7.46 | 3.00 | 3.03 | |
| Cluster 5 | 6 | | 5.97 | | 8.61 | 8.95 | | 7.67 | | 3.81 | 2.98 | 3.82 | 8.95 | 11.66 | 5.87 | |
|  | 1 | | 5.68 | | 10.31 | 16.52 | | 16.00 | | 22.25 | 15.25 | 8.51 | 16.33 | 23.42 | 22.16 | |
| Cluster 6 | 11 | | 2.79 | | 6.06 | 3.55 | | 3.50 | | 8.46 | 5.40 | 10.13 | 2.72 | 7.21 | 5.33 | |
|  | 8 | | 2.43 | | 6.41 | 1.23 | | 6.35 | | 7.51 | 5.65 | 5.61 | 5.34 | 1.11 | 1.45 | |
| Cluster 7 | 17 | | 14.46 | | 12.92 | 16.74 | | 22.37 | | 19.56 | 34.21 | 4.61 | 12.87 | 22.60 | 11.89 | |
|  | 18 | | 9.30 | | 14.48 | 6.84 | | 4.33 | | 6.52 | 18.01 | 16.52 | 14.37 | 6.11 | 4.03 | |
|  | 3 | | 31.73 | | 13.80 | 26.66 | | 16.94 | | 32.56 | 26.06 | 23.72 | 17.74 | 27.29 | 33.35 | |
|  | 4 | | 12.58 | | 9.70 | 7.52 | | 8.07 | | 2.71 | 2.83 | 2.79 | 8.43 | 6.88 | 4.04 | |
| Cluster 9 | 16 | | 6.78 | | 7.58 | 3.36 | | 12.26 | | 5.44 | 3.38 | 6.55 | 8.99 | 1.10 | 7.58 | |
|  | 2 | | 1.82 | | 15.87 | 3.49 | | 8.29 | | 13.36 | 8.38 | 13.01 | 3.41 | 5.30 | 1.66 | |
|  | |  | |  | | |  | |  |  |  |  |  |  |  |  |
|  | |  | | “Current”: use of alarms remained unchanged | | | | | | | | | | | |  |
|  | |  | | “Reduced”: use of alarms were reduced to less than 3% | | | | | | | | | | | |  |
|  | |  | | “Eliminated”: use of alarms were eliminated (0%) | | | | | | | | | | | |  |

| **eTable 2. Incident rate ratio of outcome measures between groups.** | | | |
| --- | --- | --- | --- |
| **Outcome** | **Incident rate ratios (superiority analysis, 2-tailed 95%CI)** | | |
|  | ***Current Vs. Reduced*** | ***Current Vs. Eliminated*** | ***Reduced Vs. Eliminated*** |
| Rate of falls per 1000 Occupied Bed Days | 1.02 (0.83 to 1.26), *p*=0.84 | 1.09 (0.80 to 1.49), *p*=0.59 | 1.07 (0.82 to 1.39), *p*=0.64 |
| Rate of falls-related injury per 1000 Occupied Bed Days | 1.28 (0.95 to 1.74), *p*=0.10 | 1.32 (0.95 to 1.82), *p*=0.10 | 1.02 (0.80 to 1.31), *p*=0.85 |
| Pressure injuries per 1000 Occupied Bed Days | 0.82 (0.43 to 1.57), *p*=0.56 | 0.76 (0.43 to 1.32), *p*=0.33 | 0.92 (0.63 to 1.34), *p*=0.66 |
| Medication errors per 1000 Occupied Bed Days | 0.97 (0.82 to 1.16), *p*=0.76 | 0.87 (0.68 to 1.10), *p*=0.24 | 0.89 (0.63 to 1.25), *p*=0.51 |

| **eTable 3. Monthly mean rate of alarm use across the 18 wards during the 10-month trial period.** | | | | | | | | | | | | | | | |
| --- | --- | --- | --- | --- | --- | --- | --- | --- | --- | --- | --- | --- | --- | --- | --- |
|  |  | | 2023 | | | | | | | | | | | 2024 | |
| Time | Ward | | April | | May | June | | July | August | September | October | November | December | January | |
| Cluster 1 | 14 | | 18.23% | | 0% | 0% | | 0% | 0% | 0% | 0% | 0% | 0% | 0% | |
|  | 15 | | 21.53% | | 1.32% | 1.83% | | 2.86% | 0.00% | 2.69% | 2.85% | 2.66% | 2.53% | 3.04%* | |
| Cluster 2 | 13 | | 5.68% | | 10.19% | 0% | | 0% | 0% | 0% | 0% | 0% | 0% | 0% | |
|  | 12 | | 22.80% | | 21.67% | 2.44% | | 3.14%* | 3.15%* | 3.33%* | 3.19%* | 2.73% | 3.22%* | 3.95%* | |
| Cluster 3 | 7 | | 12.12% | | 17.42% | 9.70% | | 0% | 0% | 0% | 0.40%* | 0.61%* | 0% | 0% | |
|  | 5 | | 15.63% | | 17.97% | 15.63% | | 15.63%* | 7.29%* | 8.85%* | 6.77%* | 6.51%* | 7.81%* | 5.99%* | |
| Cluster 4 | 9 | | 6.25% | | 7.81% | 10.16% | | 7.03% | 3.65%* | 0.52%* | 3.65%* | 2.43%* | 4.17%* | 9.22%* | |
|  | 10 | | 9.09% | | 3.79% | 4.24% | | 4.55% | 1.52% | 1.26% | 2.36% | 2.02% | 1.01% | 3.37%* | |
| Cluster 5 | 6 | | 2.94% | | 6.62% | 5.88% | | 2.94% | 0.00% | 0.00% | 2.21%* | 3.59%* | 0.49%* | 3.19%* | |
|  | 1 | | 8.28% | | 7.71% | 11.26% | | 9.86% | 14.09% | 2.71% | 3.32%* | 6.68%* | 5.38%* | 0.83% | |
| Cluster 6 | 11 | | 3.42% | | 3.21% | 4.62% | | 2.56% | 7.69% | 5.77% | 2.56%* | 3.13%* | 0.00% | 0.00% | |
|  | 8 | | 0.00% | | 0.00% | 0.00% | | 0.00% | 1.67% | 1.67% | 0.87% | 2.62% | 0.37% | 0.00% | |
| Cluster 7 | 17 | | 18.61% | | 30.41% | 42.50% | | 38.79% | 36.08% | 37.69% | 23.00% | 2.08%* | 0.00% | 0.00% | |
|  | 18 | | 4.58% | | 2.69% | 2.59% | | 3.04% | 6.84% | 7.77% | 6.59% | 3.63%* | 0.33% | 0.00% | |
| Cluster 8 | 3 | | 22.17% | | 21.17% | 21.05% | | 15.82% | 17.95% | 11.97% | 19.53% | 15.80% | 1.56%* | 0.00% | |
|  | 4 | | 3.33% | | 1.25% | 9.58% | | 9.38% | 3.17% | 5.58% | 13.60% | 14.80% | 0.87% | 4.67%* | |
| Cluster 9 | 16 | | 1.70% | | 0.89% | 0% | | 0.83% | 0% | 1.48% | 0% | 1.11% | 0% | 0.00% | |
|  | 2 | | 4.51% | | 2.50% | 2.50% | | 3.75% | 10.00% | 8.75% | 5.00% | 10.19% | 5.20% | 2.50% | |
|  | |  | |  | | |  |  |  |  |  |  |  |  |  |
|  | |  | | “Current”: use of alarms remained unchanged | | | | | | | | | | |  |
|  | |  | | “Reduced”: use of alarms were reduced to less than 3% | | | | | | | | | | |  |
|  | |  | | “Eliminated”: use of alarms were eliminated (0%) | | | | | | | | | | |  |
| * ward-month that did not adhere to the intervention condition. | | | | | | | | | | | | | | |  |
|  | | | | | | | | | | | | | | |  |

**
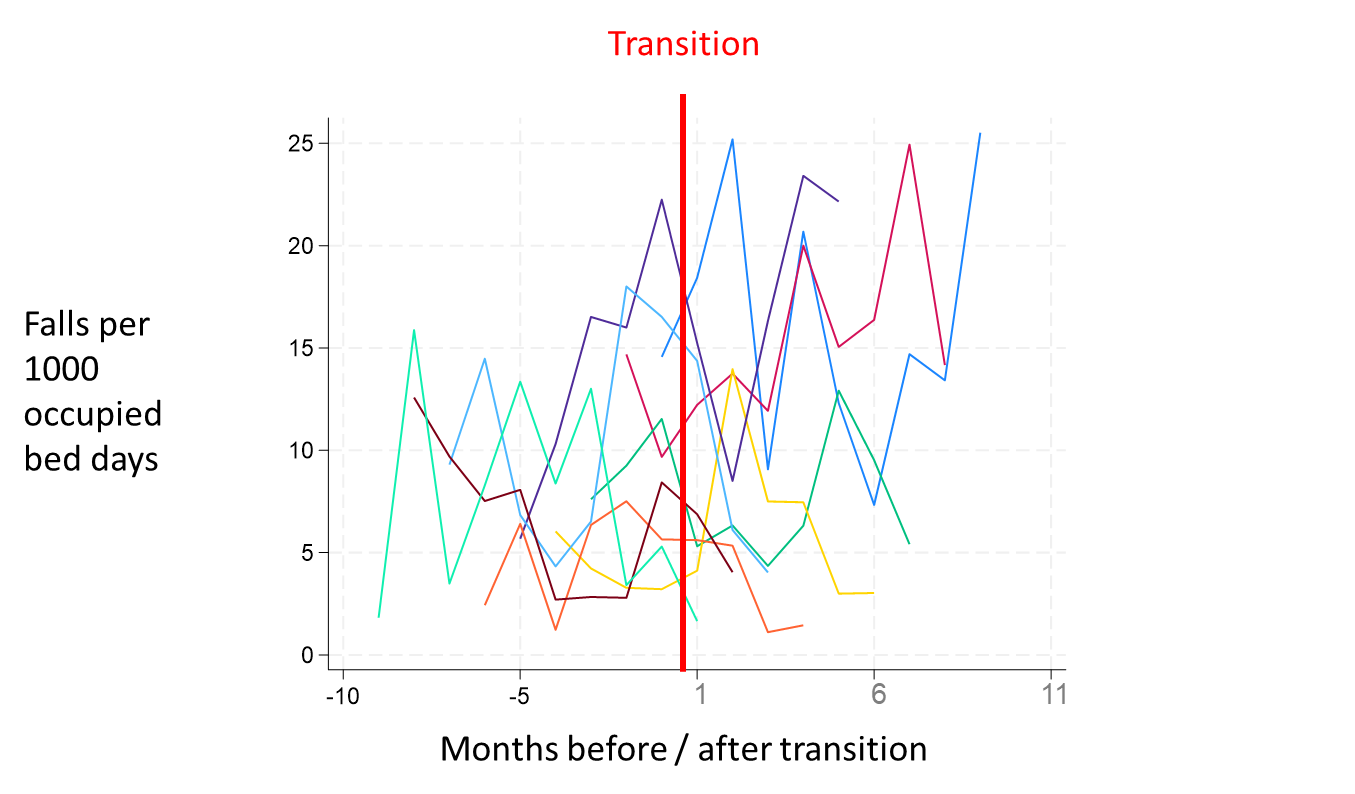
**

**eFigure 1a: Transition-relative line graph of raw number of falls for wards in the “reduced” condition.**


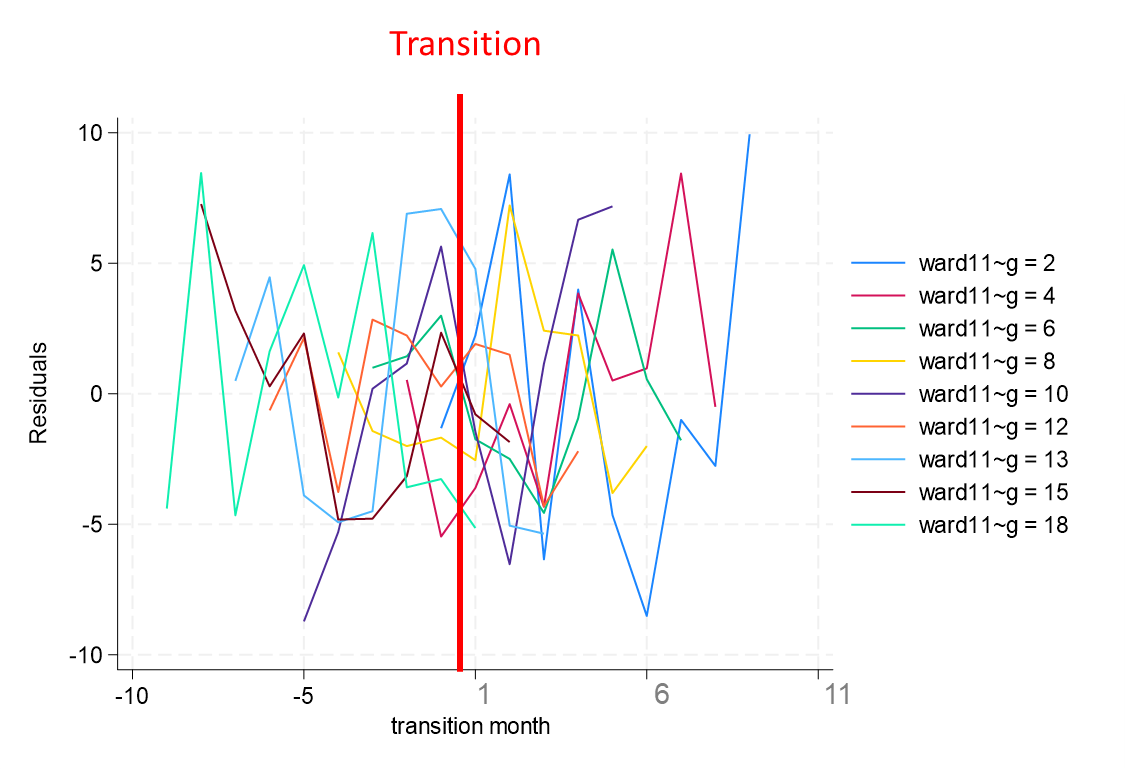


**eFigure 1b: Transition-relative line graph of residuals for wards in the “reduced” condition.**

**
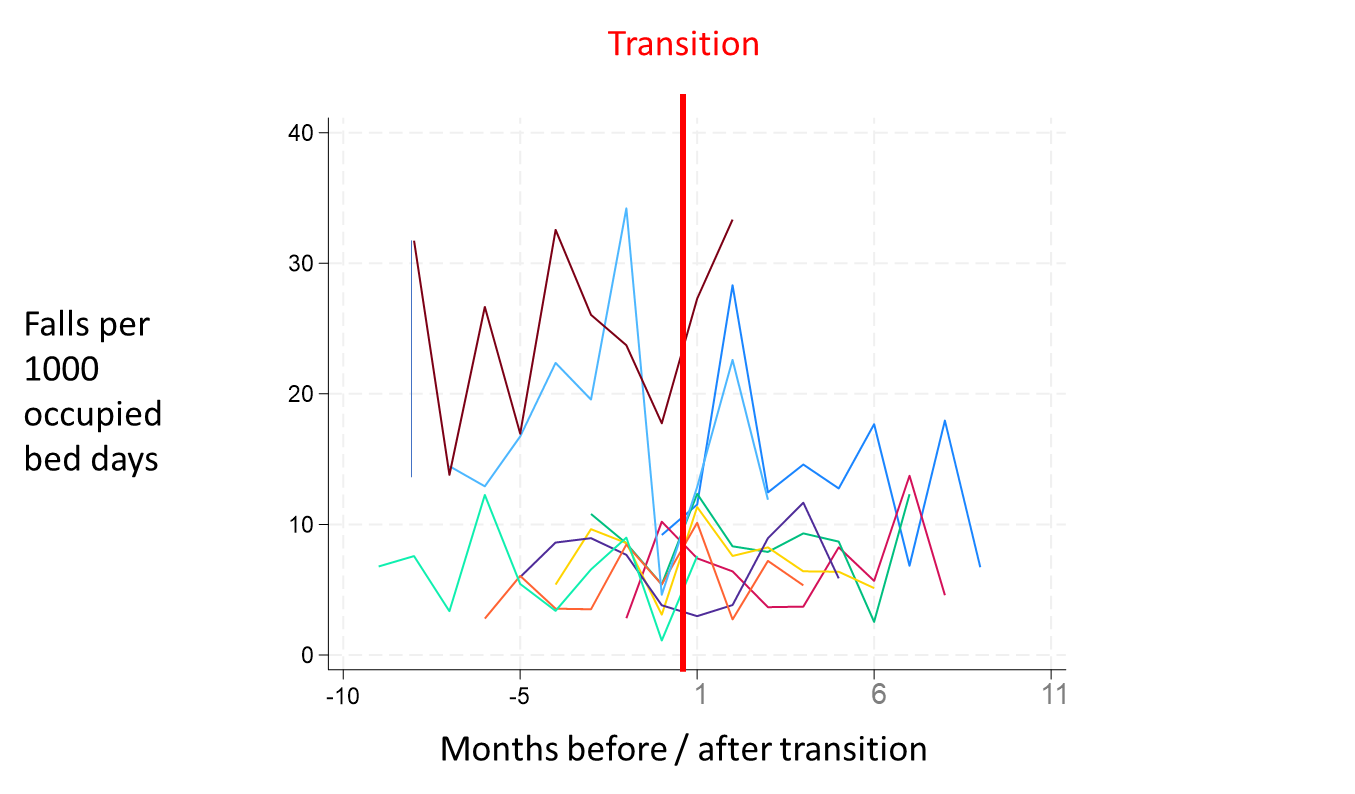
**

**eFigure 2a: Transition-relative line graph of raw number of falls for wards in the “eliminated” condition.**


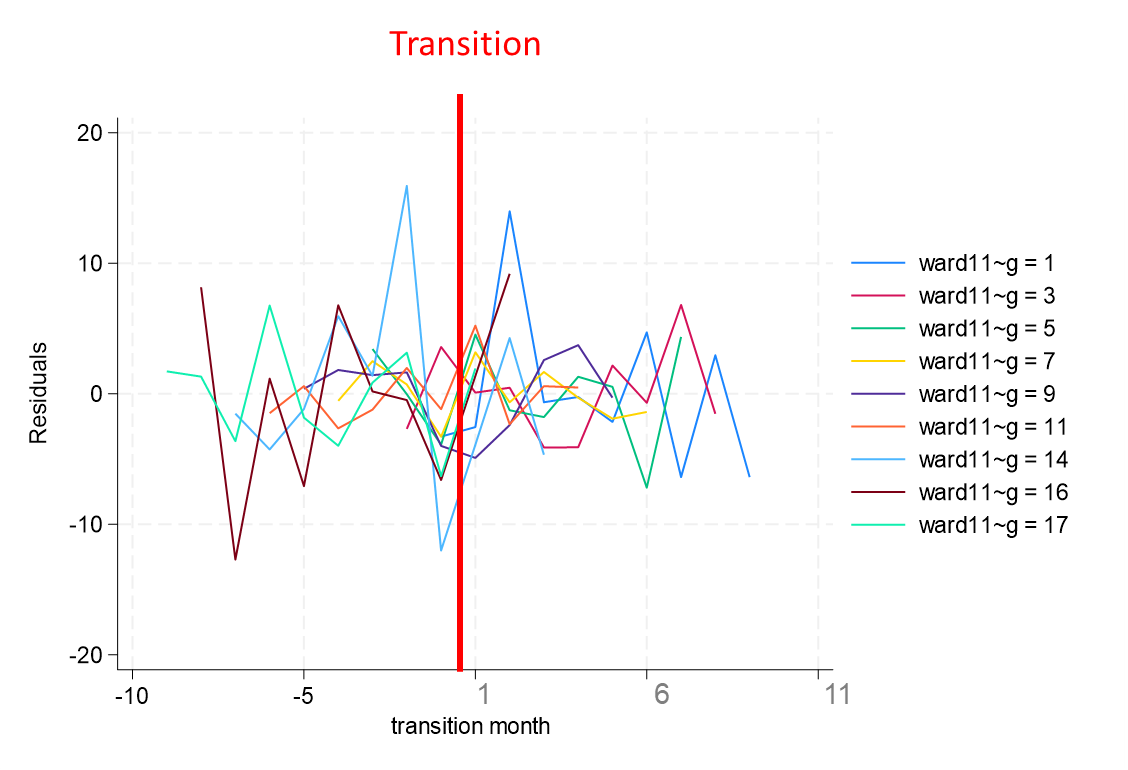


**eFigure 2b: Transition-relative line graph of residuals for wards in the “eliminated” condition.**
